# Tetra­aqua­bis[4-(methyl­amino)benzoato-κ*O*]nickel(II)

**DOI:** 10.1107/S1600536810014807

**Published:** 2010-04-28

**Authors:** Hacali Necefoğlu, Özgür Aybirdi, Barış Tercan, Yasemin Süzen, Tuncer Hökelek

**Affiliations:** a Department of Chemistry, Kafkas University, 36100 Kars, Turkey; b Department of Physics, Karabük University, 78050 Karabük, Turkey; c Department of Chemistry, Faculty of Science, Anadolu University, 26470 Yenibağlar, Eskişehir, Turkey; d Department of Physics, Hacettepe University, 06800 Beytepe, Ankara, Turkey

## Abstract

The title complex, [Ni(C_8_H_8_NO_2_)_2_(H_2_O)_4_], is centrosymmetric with the Ni^II^ ion located on a centre of symmetry. It contains two 4-(methyl­amino)benzoate (PMAB) anions and four coordinated water mol­ecules. The four O atoms in the equatorial plane around the Ni^II^ ion form a slightly distorted square-planar arrangement, while the slightly distorted octa­hedral coordination is completed by two O atoms of the PMAB anions in the axial positions. In the crystal structure, inter­molecular O—H⋯O, O—H⋯N, N—H⋯O and C—H⋯O hydrogen bonds link the mol­ecules into a three-dimensional network.

## Related literature

For structure–function–coordination relationships of the aryl­carboxyl­ate ion in transition-metal complexes of benzoic acid derivatives, see: Nadzhafov *et al.* (1981 ▶); Shnulin *et al.* (1981 ▶). For studies of transition-metal complexes with biochemical model systems, see: Antolini *et al.* (1982 ▶). For the coordination modes of benzoic acid derivatives, see: Chen & Chen (2002 ▶); Amiraslanov *et al.* (1979 ▶); Hauptmann *et al.* (2000 ▶). For related structures, see: Hökelek *et al.* (2009*a* ▶,*b* ▶); Necefoğlu *et al.* (2010 ▶); Sertçelik *et al.* (2009 ▶).
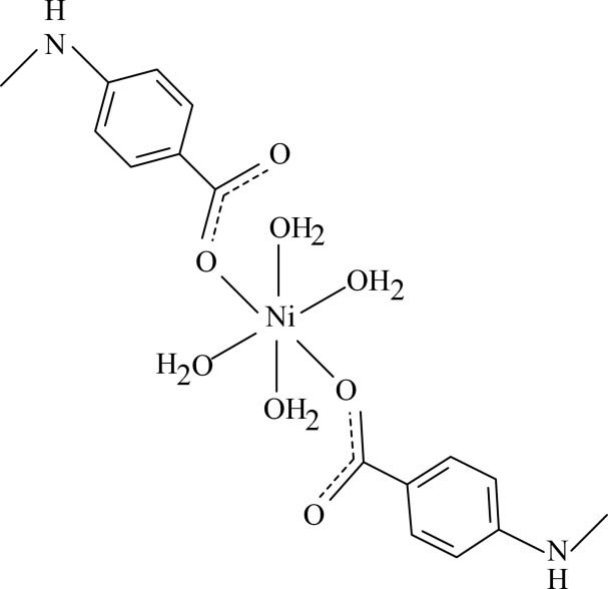


## Experimental

### 

#### Crystal data

[Ni(C_8_H_8_NO_2_)_2_(H_2_O)_4_]
*M* *_r_* = 431.06
Monoclinic, 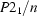

*a* = 7.5466 (2) Å
*b* = 6.1811 (2) Å
*c* = 19.4802 (3) Å
β = 98.628 (3)°
*V* = 898.40 (4) Å^3^
*Z* = 2
Mo *K*α radiation
μ = 1.13 mm^−1^
*T* = 100 K
0.35 × 0.25 × 0.13 mm

#### Data collection

Bruker Kappa APEXII CCD area-detector diffractometer
Absorption correction: multi-scan (*SADABS*; Bruker, 2005 ▶) *T*_min_ = 0.718, *T*_max_ = 0.863
8475 measured reflections
2242 independent reflections
2085 reflections with *I* > 2σ(*I*)
*R*_int_ = 0.019

#### Refinement

*R*[*F* ^2^ > 2σ(*F* ^2^)] = 0.023
*wR*(*F* ^2^) = 0.061
*S* = 1.06
2242 reflections
145 parameters
6 restraints
H atoms treated by a mixture of independent and constrained refinement
Δρ_max_ = 0.41 e Å^−3^
Δρ_min_ = −0.33 e Å^−3^

### 

Data collection: *APEX2* (Bruker, 2007 ▶); cell refinement: *SAINT* (Bruker, 2007 ▶); data reduction: *SAINT*; program(s) used to solve structure: *SHELXS97* (Sheldrick, 2008 ▶); program(s) used to refine structure: *SHELXL97* (Sheldrick, 2008 ▶); molecular graphics: *ORTEP-3 for Windows* (Farrugia, 1997 ▶); software used to prepare material for publication: *WinGX* (Farrugia, 1999 ▶).

## Supplementary Material

Crystal structure: contains datablocks I, global. DOI: 10.1107/S1600536810014807/ci5081sup1.cif

Structure factors: contains datablocks I. DOI: 10.1107/S1600536810014807/ci5081Isup2.hkl

Additional supplementary materials:  crystallographic information; 3D view; checkCIF report

## Figures and Tables

**Table 1:** Selected bond lengths (Å)

Ni1—O2	2.0537 (10)
Ni1—O3	2.0662 (10)
Ni1—O4	2.0772 (10)

**Table 2:** Hydrogen-bond geometry (Å, °)

*D*—H⋯*A*	*D*—H	H⋯*A*	*D*⋯*A*	*D*—H⋯*A*
N1—H1⋯O4^i^	0.85 (2)	2.55 (2)	3.3902 (16)	170 (2)
O3—H31⋯N1^ii^	0.91 (2)	1.97 (2)	2.8780 (16)	172 (2)
O3—H32⋯O1^iii^	0.87 (2)	1.90 (2)	2.7131 (15)	155 (2)
O4—H41⋯O1^iv^	0.96 (2)	1.68 (2)	2.6240 (14)	165 (3)
O4—H42⋯O1^iii^	0.92 (2)	2.11 (2)	2.8781 (15)	139 (2)
C8—H8*A*⋯O3^v^	0.96	2.43	3.2583 (18)	144
C8—H8*C*⋯O1^vi^	0.96	2.47	3.4105 (19)	168

Symmetry codes: (i) 
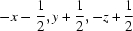
; (ii) 
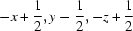
; (iii) 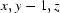; (iv) 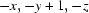; (v) 
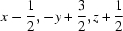
; (vi) 
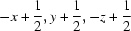
.

**Fig. 1.**
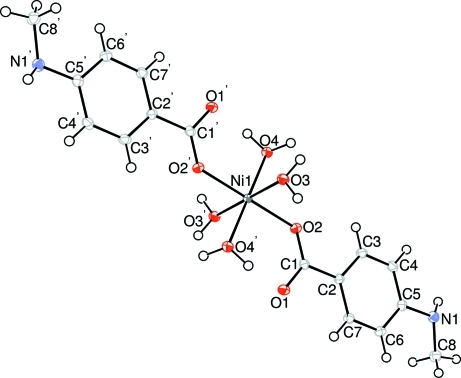
The molecular structure of the title compound, with the atom-numbering scheme. Displacement ellipsoids are drawn at the 50% probability level. The primed atoms are generated by the symmetry operation (-x, 1 - y, -z).

## supplementary crystallographic information

### Comment

The structure-function-coordination relationships of the arylcarboxylate ion in transition metal complexes of benzoic acid derivatives change, depending on the nature and position of the substituent groups on the benzene ring, the nature of the additional ligand molecule or solvent, and the medium of synthesis (Nadzhafov *et al.*, 1981; Shnulin *et al.*, 1981). Transition metal complexes with biochemical molecules frequently show interesting physical and/or chemical properties, as a result they may find applications in biological systems (Antolini *et al.*, 1982). Some benzoic acid derivatives, such as 4-aminobenzoic acid, have been extensively reported in coordination chemistry, as bifunctional organic ligands, due to varieties of their coordination modes (Chen & Chen, 2002; Amiraslanov *et al.*, 1979; Hauptmann *et al.*, 2000). The title compound was synthesized and its crystal structure is reported herein.

The title compound is a monomeric complex, with the Ni^II^ ion on a centre of symmetry. It contains two 4-(methylamino)benzoate (PMAB) ligands and four coordinated water molecules. The PMAB ligands are monodentate. The four O atoms (O3, O4, and the symmetry-related atoms O3' and O4') in the equatorial plane around the Ni atom form a slightly distorted square-planar arrangement, while the slightly distorted octahedral coordination is completed by the carboxylate O atoms (O2 and O2') of the symmetry related PMAB ligands (Fig. 1 and Table 1).

The C1—O1 [1.2761 (17) Å] and C1—O2 [1.2561 (18) Å] bonds in the carboxylate groups may be compared with the corresponding distances: 1.263 (4) and 1.249 (4) Å in [Ni(C_8_H_5_O_3_)_2_(C_10_H_14_N_2_O)_2_(H_2_O)_2_] (Sertçelik *et al.*, 2009), 1.267 (3) and 1.258 (3) Å in [Ni(C_7_H_4_ClO_2_)_2_(C_6_H_6_N_2_O)_2_(H_2_O)_2_] (Hökelek *et al.*, 2009a), 1.2616 (17) and 1.2435 (18) Å in [Ni(C_7_H_4_ClO_2_)_2_(C_10_H_14_N_2_O)_2_(H_2_O)_2_] (Hökelek *et al.*, 2009b), and 1.2678 (17) and 1.2654 (17) Å in [Ni(C_8_H_7_O_2_)_2_(C_6_H_6_N_2_O)_2_(H_2_O)_2_] (Necefoğlu *et al.*, 2010).

The Ni atom is displaced out of the least-square plane of the carboxylate group (O1/C1/O2) by 0.0728 (1) Å. On the other hand, O1, O2, N1 and C1 atoms are 0.0061 (11), 0.0549 (10), -0.0636 (13) and 0.0163 (13) Å away from the plane of the benzene ring A (C2—C7), respectively.

In the crystal structure, intermolecular O—H···O, O—H···N, N—H···O and C—H···O hydrogen bonds (Table 2) link the molecules into a three-dimensional network.

### Experimental

The title compound was prepared by the reaction of Ni(SO_4_).6(H_2_O) (1.31 g, 5 mmol) in H_2_O (50 ml) and sodium 4-(methylamino)benzoate (1.74 g, 10 mmol) in H_2_O (50 ml). The mixture was filtered and set aside to crystallize at ambient temperature for one week, giving green single crystals.

### Refinement

H atoms of NH group and water molecules were located in difference maps and refined isotropically; the O–H and and H···H distances in the water molecules were restrained to 0.95 (2) Å and 1.46 (4) Å, respectively. The remaining H atoms were positioned geometrically with C–H = 0.93 and 0.96 Å for aromatic and methyl H, respectively, and constrained to ride on their parent atoms, with U_iso_(H) = xU_eq_(C), where x = 1.2 for aromatic H and x = 1.5 for methyl H atoms.

### Crystal data

**Table:**

[Ni(C_8_H_8_NO_2_)_2_(H_2_O)_4_]	*F*(000) = 452
*M**_r_* = 431.06	*D*_x_ = 1.594 Mg m^−^^3^
Monoclinic, *P*2_1_/*n*	Mo *K*α radiation, λ = 0.71073 Å
Hall symbol: -P 2yn	Cell parameters from 5176 reflections
*a* = 7.5466 (2) Å	θ = 2.7–28.4°
*b* = 6.1811 (2) Å	µ = 1.13 mm^−^^1^
*c* = 19.4802 (3) Å	*T* = 100 K
β = 98.628 (3)°	Block, green
*V* = 898.40 (4) Å^3^	0.35 × 0.25 × 0.13 mm
*Z* = 2	

### Data collection

**Table:**

Bruker Kappa APEXII CCD area-detector diffractometer	2242 independent reflections
Radiation source: fine-focus sealed tube	2085 reflections with *I* > 2σ(*I*)
graphite	*R*_int_ = 0.019
φ and ω scans	θ_max_ = 28.4°, θ_min_ = 2.1°
Absorption correction: multi-scan (*SADABS*; Bruker, 2005)	*h* = −10→10
*T*_min_ = 0.718, *T*_max_ = 0.863	*k* = −8→8
8475 measured reflections	*l* = −25→26

### Refinement

**Table:**

Refinement on *F*^2^	Primary atom site location: structure-invariant direct methods
Least-squares matrix: full	Secondary atom site location: difference Fourier map
*R*[*F*^2^ > 2σ(*F*^2^)] = 0.023	Hydrogen site location: inferred from neighbouring sites
*wR*(*F*^2^) = 0.061	H atoms treated by a mixture of independent and constrained refinement
*S* = 1.06	*w* = 1/[σ^2^(*F*_o_^2^) + (0.032*P*)^2^ + 0.3955*P*] where *P* = (*F*_o_^2^ + 2*F*_c_^2^)/3
2242 reflections	(Δ/σ)_max_ = 0.001
145 parameters	Δρ_max_ = 0.41 e Å^−^^3^
6 restraints	Δρ_min_ = −0.33 e Å^−^^3^

### Special details

**Table:**

Geometry. All esds (except the esd in the dihedral angle between two l.s. planes) are estimated using the full covariance matrix. The cell esds are taken into account individually in the estimation of esds in distances, angles and torsion angles; correlations between esds in cell parameters are only used when they are defined by crystal symmetry. An approximate (isotropic) treatment of cell esds is used for estimating esds involving l.s. planes.
Refinement. Refinement of *F*^2^ against ALL reflections. The weighted *R*-factor wR and goodness of fit *S* are based on *F*^2^, conventional *R*-factors *R* are based on *F*, with *F* set to zero for negative *F*^2^. The threshold expression of *F*^2^ > σ(*F*^2^) is used only for calculating *R*-factors(gt) etc. and is not relevant to the choice of reflections for refinement. *R*-factors based on *F*^2^ are statistically about twice as large as those based on *F*, and *R*- factors based on ALL data will be even larger.

### Fractional atomic coordinates and isotropic or equivalent isotropic displacement parameters (Å^2^)

**Table:**

	*x*	*y*	*z*	*U*_iso_*/*U*_eq_	
Ni1	0.0000	0.5000	0.0000	0.01094 (9)	
O1	0.12315 (14)	0.95272 (17)	0.08266 (5)	0.0166 (2)	
O2	−0.00352 (13)	0.63206 (17)	0.09653 (5)	0.0153 (2)	
O3	0.23373 (13)	0.34113 (17)	0.03994 (5)	0.0150 (2)	
H31	0.327 (2)	0.418 (3)	0.0632 (10)	0.031 (5)*	
H32	0.220 (3)	0.227 (3)	0.0651 (11)	0.044 (7)*	
O4	−0.15665 (13)	0.25462 (17)	0.03223 (5)	0.0146 (2)	
H41	−0.163 (4)	0.167 (4)	−0.0089 (11)	0.070 (9)*	
H42	−0.090 (3)	0.178 (4)	0.0679 (10)	0.046 (7)*	
N1	−0.05050 (17)	1.0577 (2)	0.39282 (6)	0.0155 (2)	
H1	−0.133 (3)	0.984 (3)	0.4069 (11)	0.022 (5)*	
C1	0.04946 (17)	0.8156 (2)	0.11841 (7)	0.0130 (3)	
C2	0.02308 (17)	0.8804 (2)	0.19011 (7)	0.0126 (3)	
C3	−0.05536 (18)	0.7378 (2)	0.23236 (7)	0.0149 (3)	
H3	−0.0902	0.6007	0.2159	0.018*	
C4	−0.08175 (18)	0.7983 (2)	0.29847 (7)	0.0156 (3)	
H4	−0.1342	0.7015	0.3260	0.019*	
C5	−0.03016 (18)	1.0039 (2)	0.32435 (7)	0.0137 (3)	
C6	0.04875 (18)	1.1470 (2)	0.28224 (7)	0.0147 (3)	
H6	0.0840	1.2840	0.2987	0.018*	
C7	0.07450 (18)	1.0852 (2)	0.21607 (7)	0.0142 (3)	
H7	0.1269	1.1817	0.1885	0.017*	
C8	−0.0564 (2)	1.2874 (3)	0.41124 (7)	0.0182 (3)	
H8A	−0.0838	1.3009	0.4576	0.027*	
H8B	−0.1473	1.3589	0.3795	0.027*	
H8C	0.0578	1.3526	0.4087	0.027*	

### Atomic displacement parameters (Å^2^)

**Table:**

	*U*^11^	*U*^22^	*U*^33^	*U*^12^	*U*^13^	*U*^23^
Ni1	0.01233 (13)	0.00929 (13)	0.01089 (13)	−0.00098 (9)	0.00076 (9)	0.00030 (8)
O1	0.0221 (5)	0.0123 (5)	0.0157 (5)	−0.0032 (4)	0.0035 (4)	0.0001 (4)
O2	0.0192 (5)	0.0124 (5)	0.0140 (4)	−0.0025 (4)	0.0015 (4)	−0.0013 (4)
O3	0.0151 (5)	0.0128 (5)	0.0163 (5)	−0.0011 (4)	−0.0001 (4)	0.0012 (4)
O4	0.0162 (5)	0.0130 (5)	0.0144 (4)	−0.0015 (4)	0.0011 (4)	0.0005 (4)
N1	0.0159 (6)	0.0170 (6)	0.0143 (5)	−0.0006 (5)	0.0043 (4)	0.0006 (5)
C1	0.0118 (6)	0.0125 (6)	0.0138 (6)	0.0023 (5)	−0.0009 (4)	0.0013 (5)
C2	0.0115 (5)	0.0122 (6)	0.0134 (6)	0.0004 (5)	−0.0003 (4)	−0.0003 (5)
C3	0.0145 (6)	0.0122 (6)	0.0171 (6)	−0.0014 (5)	−0.0002 (5)	−0.0003 (5)
C4	0.0159 (6)	0.0151 (7)	0.0160 (6)	−0.0013 (5)	0.0026 (5)	0.0033 (5)
C5	0.0107 (6)	0.0158 (7)	0.0141 (6)	0.0021 (5)	0.0001 (5)	0.0008 (5)
C6	0.0143 (6)	0.0132 (6)	0.0163 (6)	−0.0014 (5)	0.0017 (5)	−0.0014 (5)
C7	0.0142 (6)	0.0134 (7)	0.0151 (6)	−0.0013 (5)	0.0021 (5)	0.0005 (5)
C8	0.0196 (6)	0.0185 (7)	0.0172 (6)	−0.0005 (6)	0.0055 (5)	−0.0018 (5)

### Geometric parameters (Å, °)

**Table:**

Ni1—O2	2.0537 (10)	C2—C3	1.3969 (19)
Ni1—O2^i^	2.0537 (10)	C3—H3	0.93
Ni1—O3	2.0662 (10)	C4—C3	1.3839 (19)
Ni1—O3^i^	2.0662 (10)	C4—C5	1.401 (2)
Ni1—O4	2.0772 (10)	C4—H4	0.93
Ni1—O4^i^	2.0772 (10)	C6—C5	1.3987 (19)
O1—C1	1.2761 (17)	C6—H6	0.93
O2—C1	1.2561 (18)	C7—C2	1.396 (2)
O3—H31	0.909 (16)	C7—C6	1.3858 (19)
O3—H32	0.873 (17)	C7—H7	0.93
O4—H41	0.964 (17)	C8—N1	1.466 (2)
O4—H42	0.926 (17)	C8—H8A	0.96
N1—C5	1.4052 (18)	C8—H8B	0.96
N1—H1	0.85 (2)	C8—H8C	0.96
C2—C1	1.4948 (18)		
			
O2—Ni1—O3	88.38 (4)	O2—C1—O1	123.87 (13)
O2^i^—Ni1—O3	91.62 (4)	O2—C1—C2	118.61 (12)
O2—Ni1—O2^i^	180.00 (2)	C3—C2—C1	120.60 (13)
O2—Ni1—O3^i^	91.62 (4)	C7—C2—C1	120.83 (12)
O2^i^—Ni1—O3^i^	88.38 (4)	C7—C2—C3	118.57 (12)
O2—Ni1—O4	85.83 (4)	C2—C3—H3	119.6
O2^i^—Ni1—O4	94.17 (4)	C4—C3—C2	120.71 (13)
O2—Ni1—O4^i^	94.17 (4)	C4—C3—H3	119.6
O2^i^—Ni1—O4^i^	85.83 (4)	C3—C4—C5	120.60 (13)
O3^i^—Ni1—O3	180.00 (5)	C3—C4—H4	119.7
O3—Ni1—O4	91.81 (4)	C5—C4—H4	119.7
O3^i^—Ni1—O4	88.19 (4)	C4—C5—N1	119.48 (13)
O3—Ni1—O4^i^	88.19 (4)	C6—C5—N1	121.60 (13)
O3^i^—Ni1—O4^i^	91.81 (4)	C6—C5—C4	118.84 (13)
O4—Ni1—O4^i^	180.00 (5)	C5—C6—H6	119.9
C1—O2—Ni1	128.50 (9)	C7—C6—C5	120.18 (13)
Ni1—O3—H31	119.5 (15)	C7—C6—H6	119.9
Ni1—O3—H32	115.3 (16)	C2—C7—H7	119.4
H31—O3—H32	106.7 (19)	C6—C7—C2	121.11 (13)
Ni1—O4—H41	96.8 (18)	C6—C7—H7	119.4
Ni1—O4—H42	109.2 (15)	N1—C8—H8A	109.5
H42—O4—H41	107 (2)	N1—C8—H8B	109.5
C5—N1—C8	118.22 (12)	N1—C8—H8C	109.5
C5—N1—H1	111.6 (14)	H8A—C8—H8B	109.5
C8—N1—H1	113.0 (13)	H8A—C8—H8C	109.5
O1—C1—C2	117.52 (12)	H8B—C8—H8C	109.5
			
O3—Ni1—O2—C1	98.00 (11)	C7—C2—C1—O2	−177.98 (12)
O3^i^—Ni1—O2—C1	−82.00 (11)	C1—C2—C3—C4	−179.27 (12)
O4—Ni1—O2—C1	−170.07 (12)	C7—C2—C3—C4	0.1 (2)
O4^i^—Ni1—O2—C1	9.93 (12)	C5—C4—C3—C2	0.0 (2)
Ni1—O2—C1—O1	−2.6 (2)	C3—C4—C5—N1	−176.96 (13)
Ni1—O2—C1—C2	176.51 (8)	C3—C4—C5—C6	−0.1 (2)
C8—N1—C5—C4	−159.10 (13)	C7—C6—C5—N1	176.96 (13)
C8—N1—C5—C6	24.13 (19)	C7—C6—C5—C4	0.2 (2)
C3—C2—C1—O1	−179.47 (12)	C6—C7—C2—C1	179.34 (12)
C3—C2—C1—O2	1.36 (19)	C6—C7—C2—C3	0.0 (2)
C7—C2—C1—O1	1.19 (19)	C2—C7—C6—C5	−0.1 (2)

Symmetry codes: (i) −*x*, −*y*+1, −*z*.

### Hydrogen-bond geometry (Å, °)

**Table:**

*D*—H···*A*	*D*—H	H···*A*	*D*···*A*	*D*—H···*A*
N1—H1···O4^ii^	0.85 (2)	2.55 (2)	3.3902 (16)	170 (2)
O3—H31···N1^iii^	0.91 (2)	1.97 (2)	2.8780 (16)	172 (2)
O3—H32···O1^iv^	0.87 (2)	1.90 (2)	2.7131 (15)	155 (2)
O4—H41···O1^i^	0.96 (2)	1.68 (2)	2.6240 (14)	165 (3)
O4—H42···O1^iv^	0.92 (2)	2.11 (2)	2.8781 (15)	139 (2)
C8—H8A···O3^v^	0.96	2.43	3.2583 (18)	144
C8—H8C···O1^vi^	0.96	2.47	3.4105 (19)	168

Symmetry codes: (ii) −*x*−1/2, *y*+1/2, −*z*+1/2; (iii) −*x*+1/2, *y*−1/2, −*z*+1/2; (iv) *x*, *y*−1, *z*; (i) −*x*, −*y*+1, −*z*; (v) *x*−1/2, −*y*+3/2, *z*+1/2; (vi) −*x*+1/2, *y*+1/2, −*z*+1/2.
